# Topical essential fatty acid oil on wounds: Local and systemic effects

**DOI:** 10.1371/journal.pone.0210059

**Published:** 2019-01-04

**Authors:** Bruno Grosselli Lania, Joseane Morari, Amanda Roberta de Almeida, Marilene Neves da Silva, Gislaine Vieira-Damiani, Karina de Almeida Lins, Carlos Lenz César, Lício Augusto Velloso, Nilson Borlina Maia, Maria Letícia Cintra, Paulo Eduardo Neves Ferreira Velho

**Affiliations:** 1 Laboratory of Applied Research in Dermatology and Bartonella Infection, School of Medical Sciences, University of Campinas, Campinas, Sao Paulo, Brazil; 2 Cell Signaling Laboratory and Obesity and Comorbidities Research Center, School of Medical Sciences, University of Campinas, Campinas, Sao Paulo, Brazil; 3 National Institute of Photonics Applied to Cell Biology (INFABIC), Gleb Wataghin Institute of Physics, University of Campinas, Campinas, Sao Paulo, Brazil; 4 Clinical Medicine Department, School of Medical Sciences, University of Campinas, Campinas, Sao Paulo, Brazil; 5 Aromatic Plants Division, Agronomic Institute of Campinas, Campinas, Sao Paulo, Brazil; 6 Pathological Anatomy Department, School of Medical Sciences, University of Campinas, Campinas, Sao Paulo, Brazil; US Army Institute of Surgical Research, UNITED STATES

## Abstract

**Background:**

The use of medicinal plants and their derivatives is increasing, and approximately one-third of all traditional herbal medicines are intended for wound treatment. Natural products used in these treatments include vegetable oils, which are rich in essential fatty acids. Once in contact with an ulcerative surface, the oil reaches the blood and lymphatic vessels, thus eliciting systemic effects.

**Objective:**

This study evaluated the local and possible systemic effects of essential fatty acids (sunflower oil) applied topically to rat wounds.

**Methods:**

Cutaneous punch wounds (6 mm) were produced on the dorsa of 30 rats. Saline (SS), mineral oil (MO) or essential fatty acid (EFA) solutions were applied topically. Healing was evaluated after 2, 4 and 10 days (n = 5 per group) by visual and histological/morphometric examination, second harmonic generation (SHG) microscopy, and cytokine and growth factor quantification in the scar tissue (real-time PCR) and in serum (ELISA).

**Results:**

MO/EFA-treated animals had higher IGF-1, leptin, IL-6 and IFN-γ mRNA expression and lower serum IL-6 levels than the control (SS/MO) animals. SHG analysis showed no difference in collagen density between the animals treated with MO and EFA.

**Conclusion:**

EFA treatment induces topical (observed by local IGF-1, leptin, IL-6 and IFN-γ production) and systemic effects, lowering IL-6 levels in the serum. As the oil is widely used to shorten ulcer healing time, studies are needed to evaluate the treatment safety and possible undesired effects.

## Introduction

The wound healing process encompasses four highly integrated and overlapping phases: hemostasis, inflammation, proliferation, and tissue remodeling [1]. The molecular complexity of the wound repair process stems from the fact that it is modulated by hundreds of genes involved in growth factor, pro-inflammatory cytokine and chemokine signaling [2]. The skin is now recognized as an endocrine organ, which can produce hormones, express different receptors and also modulate local cytokine production [3].

Plant-based strategies and herbal drugs are alternative approaches to wound treatment, mainly in developing countries, because of their simplicity and low cost [4, 5]. Almost 65% of the world’s population has used plants as medicinal agents in primary healthcare modalities, and approximately one-third of all traditional herbal medicines are intended for treating wounds or skin disorders compared with only 1–3% of modern drugs [2].

Regarding medicinal plant extracts, oils are mainly used for skin healing purposes, especially those rich in essential fatty acids (EFAs), because these compounds (such as linoleic acid) are necessary for maintaining epidermal integrity and the skin’s water barrier [6]. Sunflower seed oil contains lipids similar in composition to the stratum corneum [7] and is one of the most applied oils that can be used in a regular diet [8]. However, the safety of these oils must be tested, as they may induce an inflammatory response [9]. In Brazilian hospitals, it is common to apply EFA-embedded gases on wounds (such as pressure ulcers) [6].

We assessed the local and possible systemic effects of topical application of an EFA-rich oil on rat skin wounds.

## Materials and methods

Thirty female Sprague-Dawley rats (strain NTacUnib:SD), with a median age of 7 weeks, were obtained from the University of Campinas Breeding Center (*CEMIB*). The study was approved by the University of Campinas Institutional Animal Care and Use Committee (IACUC) under protocol number 2929–1 and was conducted in accordance with the guidelines of the Brazilian College for Animal Experimentation and the International Council for Laboratory Animal Science (ICLAS). To follow the IACUC recommendations for using the lowest possible number of animals per group and to evaluate the systemic action of topically applied EFA oil (Saniplan, Rio de Janeiro, RJ, Brazil), we used a model in which the control group animals received two treatments [10]: saline solution as a control and mineral oil (MO) as a treatment. The treatment group also received two topical agents: MO as the control and EFA oil as the treatment. The chemical content of the sunflower oil is as follows: capric acid, caprylic acid, caproic acid, lauric acid, linoleic acid, lecithin, retinol palmitate, tocopherol acetate and alpha-tocopherol [11]. The mineral oil used was manufactered by Rioquímica (São José do Rio Preto, São Paulo, Brazil) and is made of liquid petrolatum. This oil is used to maintain the skin moist, by avoiding transepidermal water loss (TEWL). The petrolatum in not absorbed percutaneously; the absorption is predominant in the stratum corneum [12].

According to Petry and colleagues, mineral oil is a mixture of predominantly saturated hydrocarbons consisting of straight-chain, branched and ring structures with carbon chain lengths primarily greater than C16, often referred to as ‘MOSH’ (mineral oil saturated hydrocarbons [12]. This oil was used as control because it has the capacity to keep local moisture, comparing to the saline solution. In this way, it was possible to analyze the actions of sunflower oil, excluding the possible benefits that even a inert oil could bring to the wound healing process [13].

**Fig 1:**
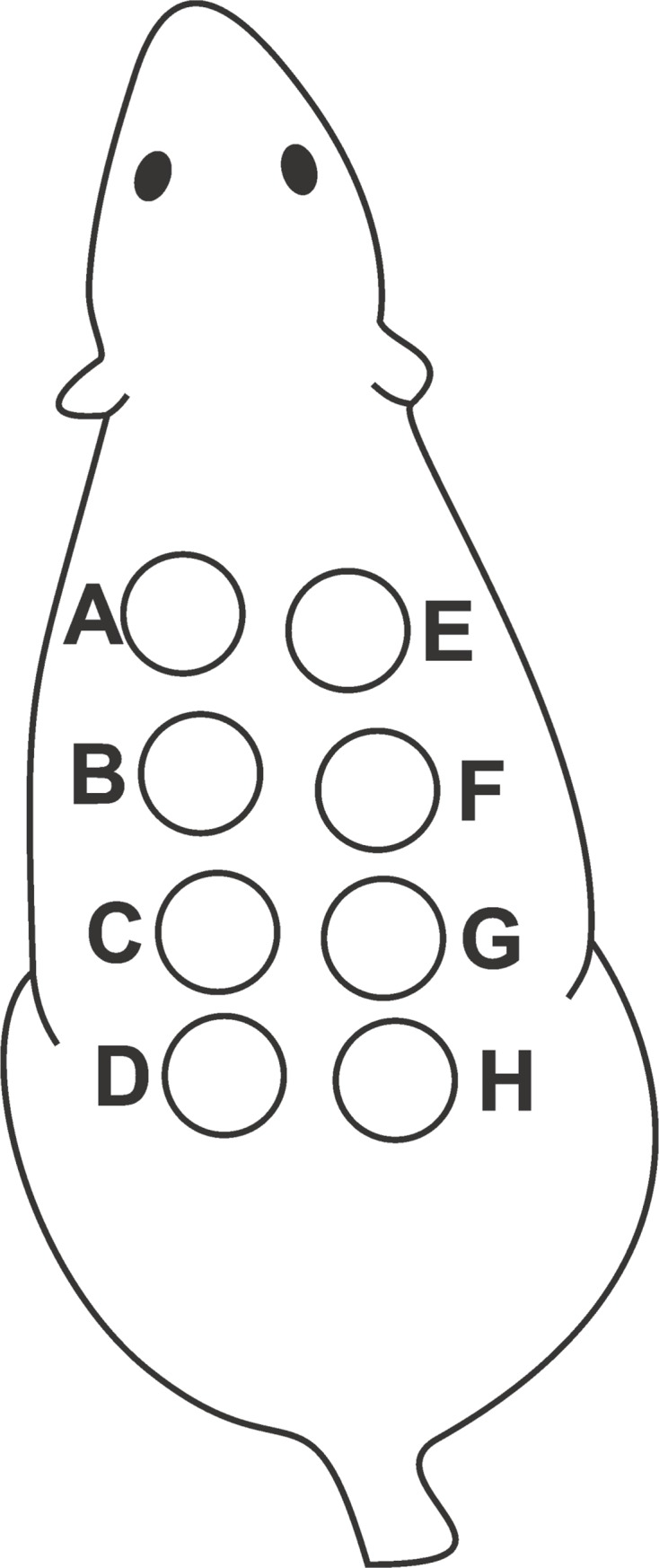
Scheme of the wounds produced on the dorsum of each animal. Lesions on the left side (A-D) received the control agent: SS in group 1 and MO in group 2. Lesions on the right side (E-H) received the treatment: MO in group 1 and EFA in group 2.

The animals were housed in individual polypropylene cages with free access to food and water. Room temperature was set at 22°C, and lighting was set on a 12:12-hour artificial light-dark cycle. The rats were anesthetized with a combination of ketamine at 50 mg/kg^-1^, xylazine at 7.0 mg/kg^-1^ and diazepam at 2.0 mg/kg^-1^. Using 6.0-mm biopsy punches, we produced eight wounds on the back of each animal: four on the left side and four on the right side. The animals were randomly divided into 2 groups of 15 animals as follows: group 1 –control (SS/MO) in which wounds were only cleansed with saline solution (SS) on the left side and treated with mineral oil (MO) on the right, and group 2 –(MO/EFA) in which lesions received MO on the left and treated with EFA oil on the right (Fig 1). After the wounds were produced, the animals received the specific treatment for each lesion. Lesions were covered with gauze and tape to avoid mixing of the different oils applied to the same animal. All animals were followed up after the procedure until the end of the anesthesia, to find out how they would wake up and to get food and water. To diminish/control pain, animals had access to water with paracetamol (200 mg.kg^-1^) *ad libitum* on the first two days and were evaluated twice a day for signals of distress (low water and food intake, excessive vocalization, weight, facial expressions [14]). All wounds were cleansed daily with SS, the treatments were reapplied, and the dressings were replaced until the animals were euthanized.

### Sample collection

After random selection for euthanasia via cardiac puncture on days 2, 4, and 10 (five animals each day), the rats were anesthetized with thiopental at 85.0 mg/kg^-1^ and lidocaine at 10 mg/ml (in order to confirm the deep anesthesia, the animals’ had their breathing rate and lack of response to stimuli)[15, 16] photographs of the lesions were taken at a fixed height, and blood was immediately collected for serological analyses via ELISA. Skin fragments from the wound area were excised to the fascia and bisected: half for histological analyses (wounds closer to the animal’s head, two for each treatment type) and the other half for real-time PCR analyses (wounds closer to the animal’s tail).

### Clinical evaluation

During daily care, the wound contraction rate, decreases in erythema and edema and re-epithelization were visually assessed by comparing the lesions with each other and with the previously taken photos.

### Wound area

Wounds were photographed on days two, four and 10 after surgical intervention with a constant focal length. The unhealed areas were quantified by the “number of pixels”. The reduction relative to the topographic opposite (on the left side) was determined for each wound treated (on the right side). In this way, the proportion of the treated wound that remained unhealed after the experiment began could be quantified in relation to the control side of the same animal. This proportion was considered 100% on day zero. [17, 18]

### Histology

H&E-stained skin sections from all animals were evaluated by a dermatopathologist for qualitative analysis of the cells, such as fibroblasts, endothelial cells, and lymphomononuclear cells, amid collagen bundles (mature granulation tissue). For morphometry (performed in ten x400 random fields using an eyepiece with a cycloids grid coupled to the ocular lens), all nuclei bordering the cycloids were counted, and the results were recorded as the average number of cells in the scarred area, as previously described [19].

### Second harmonic generation (SHG) analysis

We used nonlinear imaging to evaluate collagen density in the skin via the SHG technique. Wound areas images were obtained at the National Institute of Photonics Applied to Cell Biology (INFABIC) using a Zeiss Axio Observer.Z1 LSM780 microscope (Carl Zeiss, Oberkochen, Germany) with 512 × 512 pixels of spatial resolution. A MaiTai Ti:Sapphire laser (Spectra Physics, Santa Clara, CA, USA) was used to excite the samples at 800 nm with a 10x objective lens. For quantitative image analyses, we used the ImageJ optical density tool (available from http://rsb.info.nih.gov/ij). The wound center was selected as the specific region of interest (ROI), and the same box (with the same area) was used in images obtained from animals euthanized on day 10.

### RNA extraction

Newly-formed healed skin samples (scar tissue) were obtained using 6-mm punches of the same size and in the same location as the original lesion to avoid collecting normal skin [18]. By comparing in this way, the differences presented in this manuscript are strictly due to the treatment.

Skin samples were homogenized in 1 mL of TRIzol (Invitrogen, Carlsbad, CA, USA), and total RNA was obtained according to the manufacturer's instructions. A High Capacity cDNA Reverse Transcription Kit (Life Technologies, Carlsbad, CA, USA) was used to reverse transcribe three micrograms of total mRNA.

### Real-time PCR

To perform PCR, we used 40 ng of cDNA, 0.5 μl of specific primer, 5.0 μl of Taqman Universal Master Mix (4369016, Life Technologies, Carlsbad, CA, USA) and RNase-free water at a final volume of 10 μl. TaqMan gene expression analyses were performed on a Step One Plus Real-Time PCR System (Applied Biosystems, Foster City, CA, USA). The reference gene was rat GAPDH, cat. number 4352338E (Applied Biosystems, Foster City, CA, USA).

The target gene primers were purchased from Applied Biosystems and included the following: adiponectin (Rn00595250_m1), leptin (Rn00565158_m1), IL-2 (Rn00587673_m1), IL-4 (Rn01456866_m1), IL-6 (Rn01410330_m1), IL-12 (Rn00575112_m1), IFN-α (Rn01400027_g1), IFN-γ **(**Rn00594078_m1), IGF-1(Rn00710306_m1), and TNF-α (Rn00562055_m1). The control group (MO) average on each treatment day (day 2, 4 or 10) was used to calibrate all groups. PCR efficiency was calculated prior to the experiments, and the 2^-DDCT method was used to calculate gene expression [20].

### ELISA

Serum samples from all animals were obtained via whole blood centrifugation (10 min, 4°C, 3,500 RPM). We used Quantikine Quantitative Sandwich Enzyme Immunoassay ELISA kits for IL-2, IL-4, IL-12, IL-6, IGF-1, IFN-α, IFN-γ, and TNF-α (R&D Systems, Minneapolis, MN, USA) and for leptin and adiponectin (Millipore, St. Charles, MO, USA). All plates were analyzed using a BioRad 680 microplate reader, (Bio-Rad Laboratories, Hercules, CA, USA) at 450 and 570-nm wavelengths, according to the manufacturers’ recommendations.

### Statistical analysis

All data were analyzed using GraphPad Prism software. Two-way analysis of variance (ANOVA) and Bonferroni’s post-test at a significance level of 5% were used to determine statistically significant differences.

## Results

### Clinical evaluation

During daily treatment (dressing changes, cleansing and reapplication of treatments), the animals were visually evaluated to assess the wound healing process.

**Fig 2:**
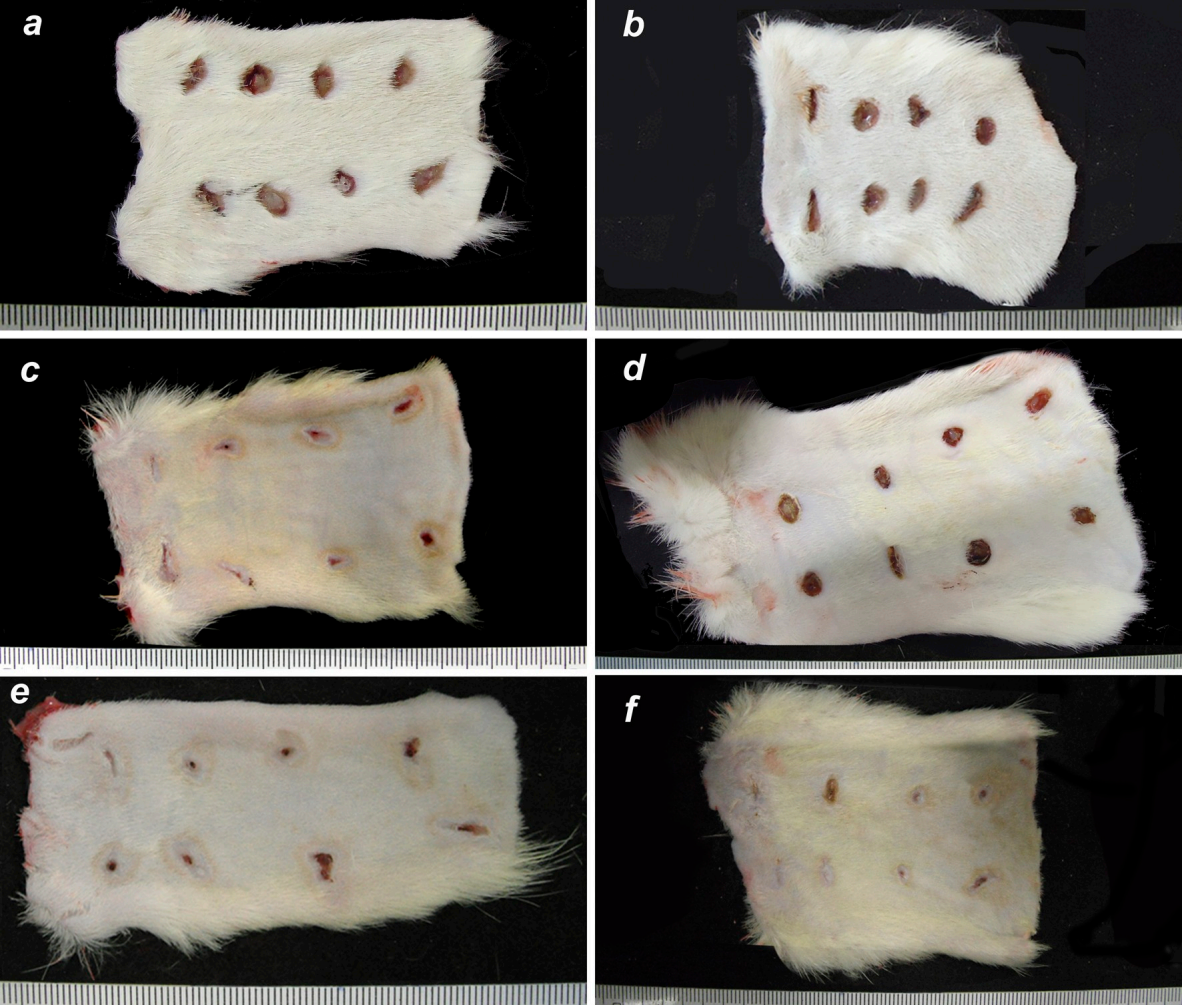
Punch wounds, back skin of the rats. Control group on the left and topical essential fatty acid solution treatment on the right (a,b: day 2; c,d: day 4; e,f: day 10). The treatment that each lesion received is in accordance with Fig 1. Scale in millimeters.

Throughout the experiment, the wounds treated with MO and EFA healed better than those treated with SS. The use of EFA provided a better clinical outcome than MO. On Fig 2 bellow we show representative images of the animals’ excised skin with the wounds.

### Wound area

It was found that the treatment with EFA-rich oil had a slower wound healing outcome (Table 1). It was needed 7.2 days (n = 5) to achieve 25% of healed area and around 10 days to achieve 50% of total healed area.

**Table 1:** Days needed to reach 25 and 50% of total area healed using SS or EFA oil.

Treatment	Healed area		Δ
	25%	50%	
SS	4.72	7.7	0.422
EFA	7.2	10.0	0.738

### Histology

**Fig 3:**
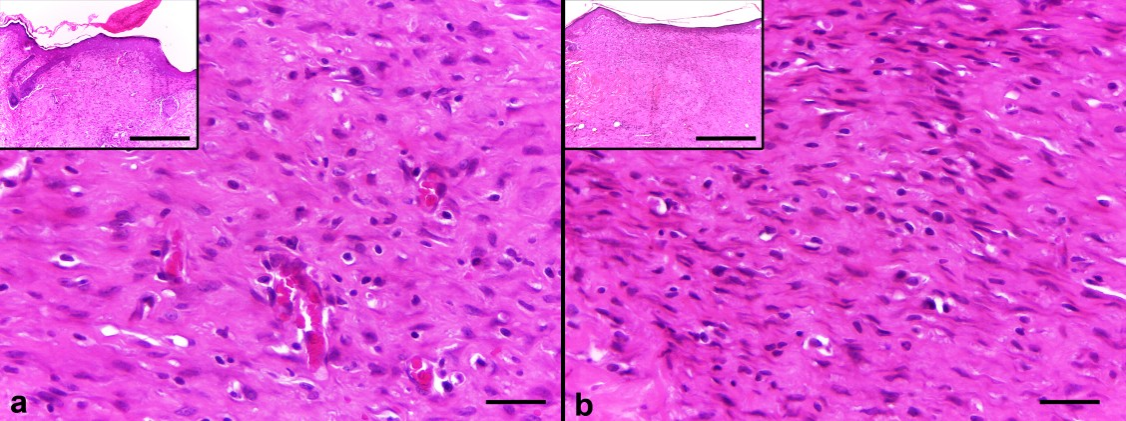
**Comparative histological view of EFA-treated lesions (a) and MO-control lesions (b) on day 10**. Complete re-epithelialization and a low amount of cellular granulation tissue are seen with evident extracellular matrix deposition (H&E, original magnification x500 and x120 [insets]).

Wounds that received SS had more cells then all at day 10. We observed less cellular density in the wounds treated with EFA compared with those treated with MO in the control group. This finding is consistent with the clinical evaluation described above (Fig 3).

**Fig 4:**
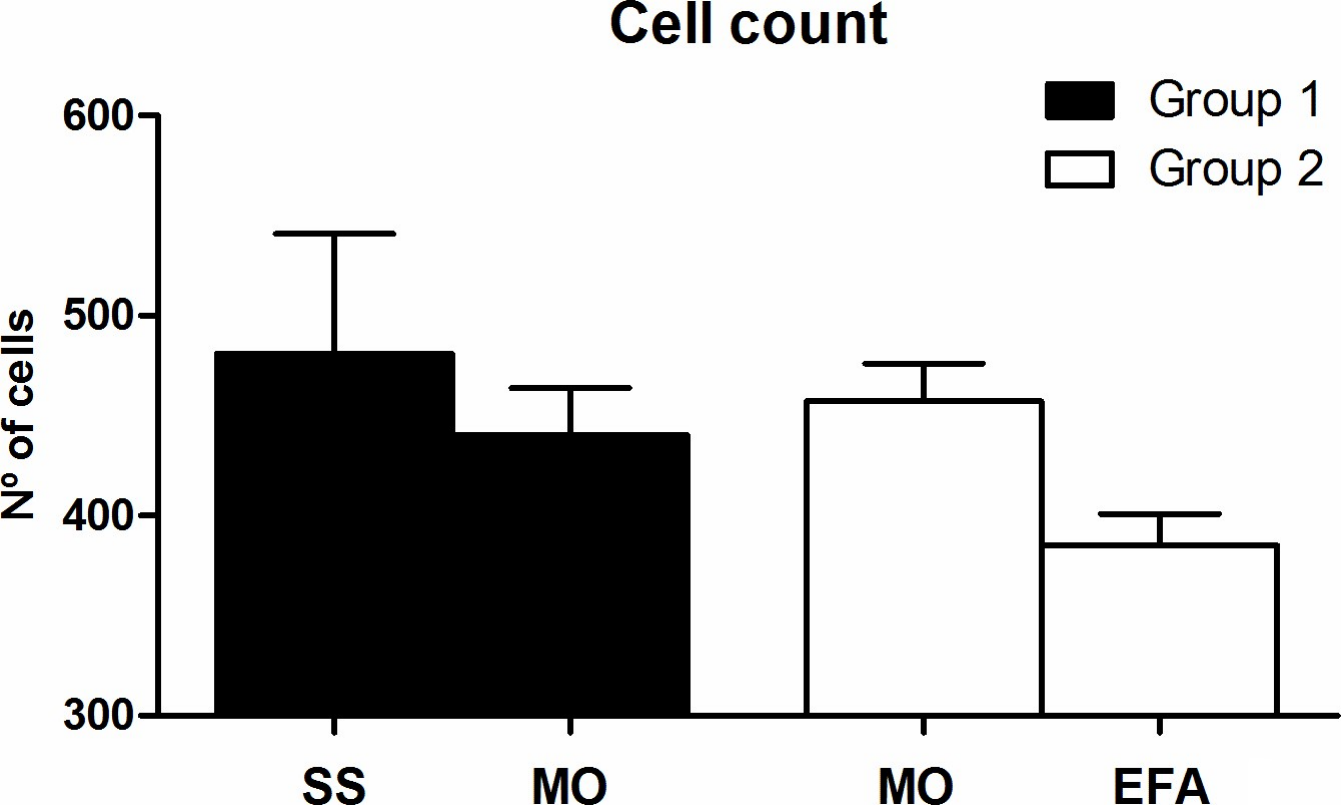
**Graph of the cell number**: Showing the average number of cells in the SS/MO and MO/EFA groups for both the control and treated wounds on day 10.

Although the number of cells decreased (corresponding to a relatively higher collagen density) in the wound bed on day 10 in the MO/EFA group compared with the SS/MO group (Fig 4), showing a strong tendency to difference between these treatments, the sides and groups did not statistically differ.

### SHG

**Fig 5:**
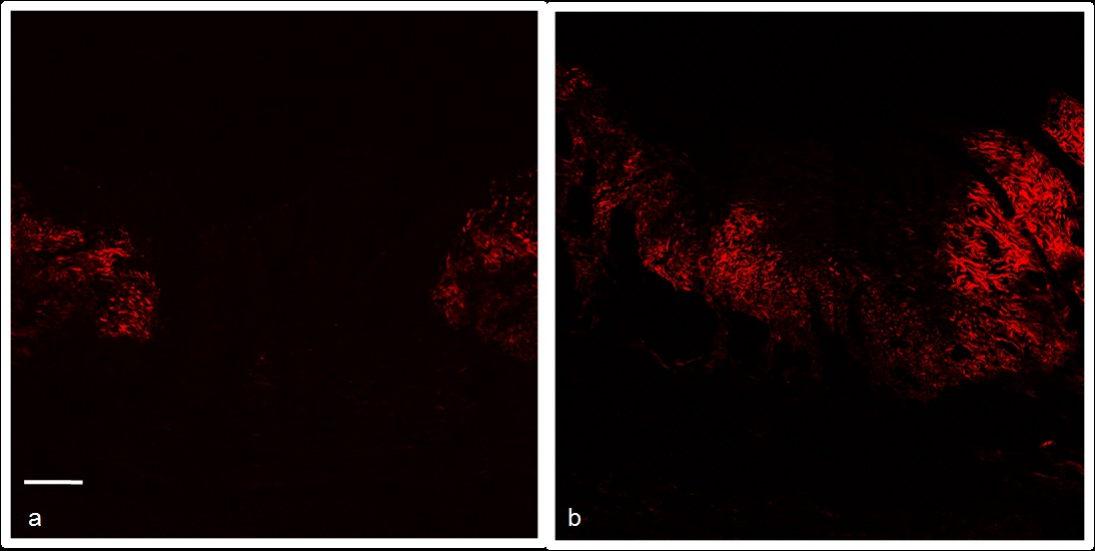
Representative images of the SHG histology photomicrographs used to evaluate collagen density. SS-treated lesions (control group) (A) and EFA-treated lesions (B).

By comparing the treated with the control wounds in both groups (Fig 5), the SHG analysis of the collagen density revealed no significant difference between the groups. The medium density value found for the SS-treated lesions group was 14,853.78 vs. 17.951,98 of the EFA-treated group (n = 5).

### Real-time PCR

**Fig 6:**
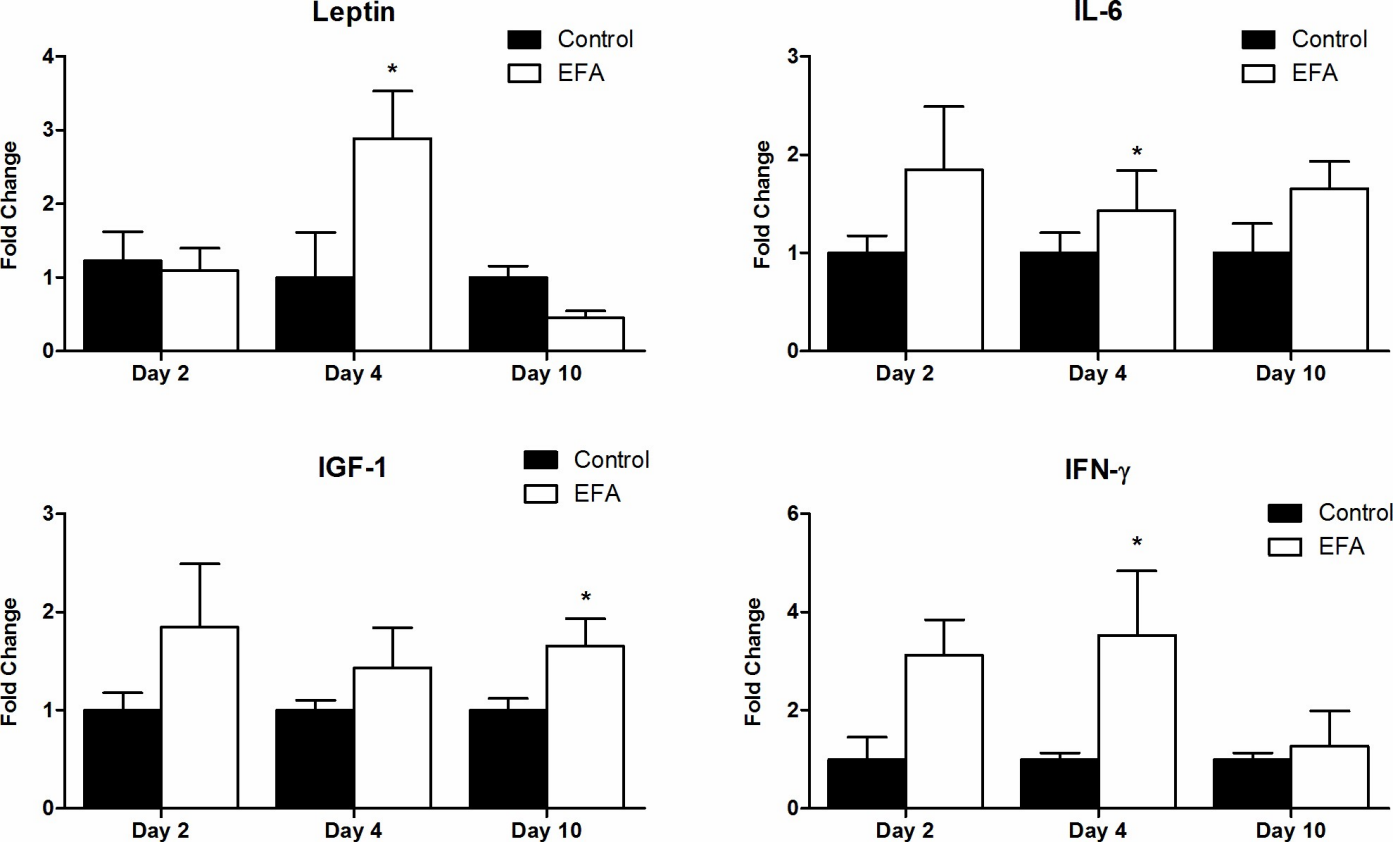
Graph of the qPCR results. Presenting the leptin (p<0.05), IL-6 (p<0.01), IFN-γ (p<0.05) and IGF-1 (p<0.05) mRNA levels; “*” at the top of the bars indicates which analysis revealed a significant difference compared with the control. All data are expressed as the number of fold-changes.

Our qPCR results (Fig 6) showed that the EFA-treated lesions in group 2 animals displayed increased levels of leptin (2.88±0.65 vs. 1.00±0.61-fold change), IL-6 (2.84±0.575 vs. 1.00±0.206-fold change) and IFN-γ (3.53±1.31 vs. 1.00±0.13-fold change) in the initial stages (day 4) of wound healing compared with the MO-treated lesions in the control group. IGF-1 levels were higher on day 10 in the EFA-treated animals (2.15±0.325 vs. 1.00±0.118-fold change). Analyses of the remaining cytokine values were not significant. No statistical differences were found between SS-treated lesions and MO-treated ones.

### ELISA

**Fig 7:**
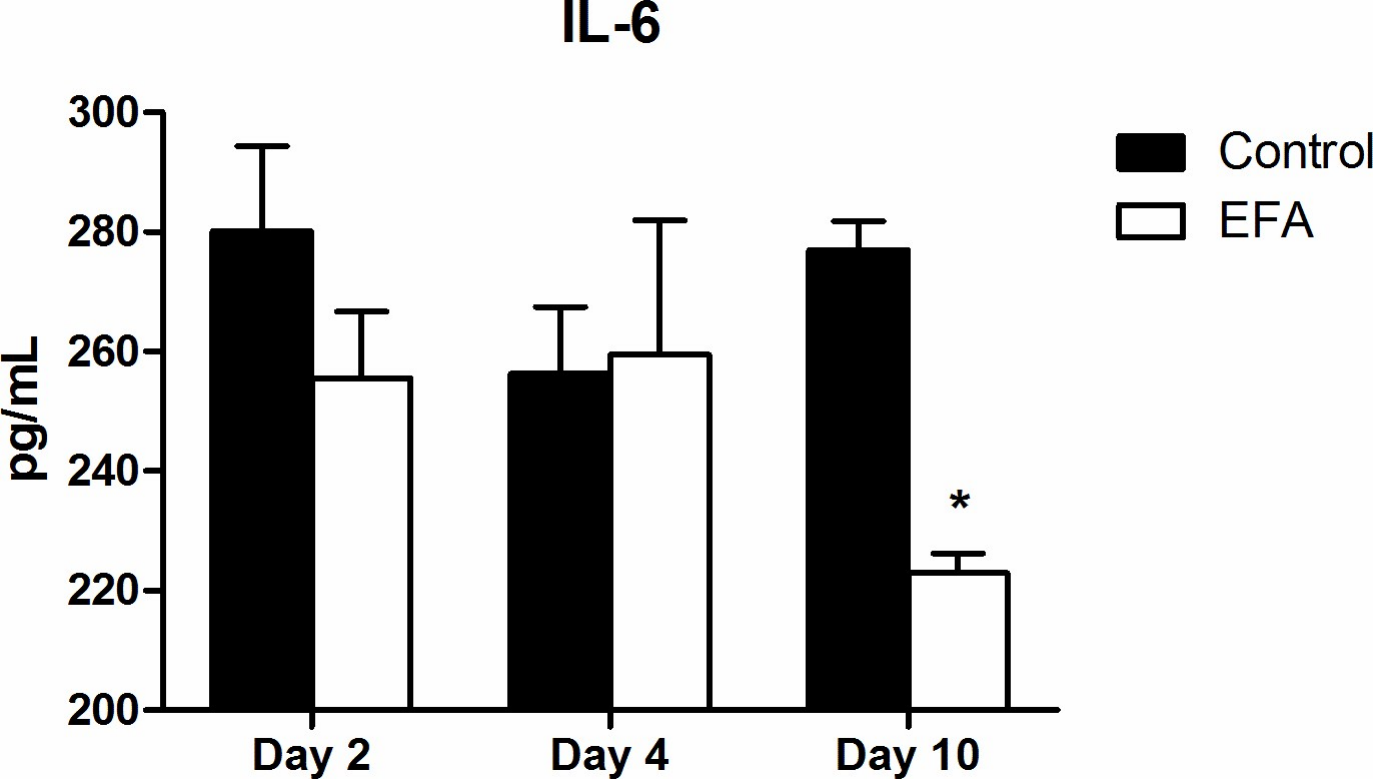
Graph of the ELISA results. Showing IL-6 serological levels in the control and EFA groups on day 10 (p<0.05); “*” at the top of the bar indicates the analysis that showed a significant difference compared with the control.

When analyzing the serological cytokine levels (Fig 7), we found significant differences between groups only in IL-6 levels: the animals treated with EFA had lower IL-6 expression on day 10. No other cytokine analyses revealed significant values.

## Discussion

Using EFA in large volumes to treat cutaneous wounds and the scarcity of scientific information on its efficacy and safety prompted us to objectively study its topical and systemic effects [21–25].

Although the results of the histomorphometric examination favored EFA-rich oil to accelerate the healing process, the differences were not significant. A larger cohort may reveal statistically significant results. The contrasting results found in wound area measurement can be explained by the presence of the crusts. The photographs were taken before its removal from the scar tissue, what could have led to worse results compared to the control group [18].

The cytokine levels detected in the tissue samples (measured via PCR) were expected because this oil is reported to induce local results [26]. These findings were expected because the EFA oil contains essential lipids needed by cells [27], and improved wound healing has been reported when this product is used.[28, 29]

We found statistically significant results with regard to local leptin, IL-6, IGF-1 and IFN-γ mRNA levels. A study performed by Tadokoro and colleagues revealed that leptin applied at the wound site accelerated wound healing by promoting keratinocyte proliferation [30]. This adipokine triggers responses mainly in the hypothalamus but also in other cell types that have leptin-receptors, such as endothelial cells, keratinocytes, and fibroblasts [31]. Moreover, this study also found high local leptin levels in samples of keloid and hypertrophic skin lesions compared with normal skin samples [31]. Our results are consistent with those presented in Tadokoro’s research. An interesting finding of our research is the lower levels of this adipokine mRNA at later healing stages, which may have prevented exaggerated wound healing responses.

IGF-1 can be produced by fibroblasts and may induce mitogenic and motogenic responses in keratinocytes [32]. Thus, our results indicated that wounded skin treated with EFA had higher local IGF-1 mRNA expression throughout the experiment, which may benefit the wound healing outcome. Gene therapy with the IGF-1 gene transferred to fibroblasts was found to upregulate the expression of IGF-1 and improve wound healing outcome [33].

IFN-γ mRNA levels were higher in animals treated with EFA in our experiment. A previous study found that this substance delayed the wound healing process in burn injuries and that knockout mice exhibited better angiogenesis and myofibroblast responses due to a dampening of excessive inflammation [34]. Our results showed the opposite response; the EFA-treated group had better wound healing but higher IFN-γ mRNA levels. This can be explained by the other cytokines involved in the wound healing cascade, which may have overlapped with the effects of interferon. In addition, its higher levels may have been related to the high cellularity our group found at day 10.

Cytokines produced by the skin may have access to capillary vessels through diffusion, even though the rate of diffusion depend on factors such as degradation level, distance between production and the vessels [3]; one of these cytokines produced in the skin is IL-6 [35, 36]. The significant changes in the serum IL-6 levels provided evidence that topically applying EFA-rich oil produced a systemic effect. This is a new and important finding that demonstrates the systemic effects of topical application of this oil by comparing the serum level of cytokines in MO/EFA-treated mice with those in SS/MO-treated mice. As it is largely used in open wounds regardless of their size and in some cases during the entire treatment, there is a risk of systemic adverse side effects. IL-6 is a pro-inflammatory cytokine that also coordinates an anti-inflammatory environment to resolve inflammation [37]. High IL-6 levels are directly related to rheumatoid arthritis, diabetes [38], depression [39], and myeloma, leading to a medical interest in controlling IL-6 activity, since it can act in tumor cell growth [40]. Low IL-6 levels were found in patients with IgA nephropathy [41]. Moreover, IL-6 is putatively involved both in skin burns [42] and excisional wound healing [43, 44]. In both cases, higher IL-6 levels at initial healing stages were associated with better outcomes. Goodman and Stein [45] reported that aging fibroblasts produced lower IL-6 levels, which in older persons could lead to poor wound healing and infections [45]. This same scenario could be induced by extensive EFA oil when topically used for wound healing, thus worsening the process [11]. Another study found that animals fed with an EFA oil-enriched diet had lower systemic IL-6 levels than the control group [46].

Our model allowed observation of cytokine and growth factor production between the groups, as well as the cellularity level both between groups and between different treatments in the same group. We found increased IL-6 expression at day 4, in the beginning of the wound healing process and a systemic downregulation at day 10. In a work performed by Wiik and colleagues, it was found an opposite situation: higher local levels and lower systemic levels [47]. This is a new and important finding made by our research. This may help to prevent hypertrophic scars [48]. Since the animals of all groups were kept at the same conditions throughout the experiment, we may exclude an external cause for this discrepancy. As cited above, higher levels of IL-6 at the early stages of wound healing is positive, as well as decreased levels at later stages. In order to thoroughly assess the role of IL-6, further studies should be performed, also for other cytokines enrolled in the process that we could not detect statistical differences.

An important feature to assess in the use of oils to enhance wound healing is the effect of the vehicle used in the formulation, since it can can influence the active ingredient effects [49]. In a previous research performed by our group, we found that using coffee oil grounds worsened the wound healing outcome, due to the formation of granulomas [18]. In the present work, we used a commercial sunflower oil (Saniplan, Rio de Janeiro, Brazil).

EFA oil has been approved by the Brazilian Health Regulatory Agency (ANVISA) as a curative/dressing; however, no scientific literature is available for public consult regarding its safety. A few articles exist on the use of EFA on skin wounds in rats [50–52], lambs [53] and human patients [22], but none of these evaluated local or serum changes in the cytokines involved in the wound healing cascade. Two review articles stated that no contraindications exist for directly applying EFA oil to a wound surface [28, 54], but both articles lacked information regarding the systemic effects. Further studies should be performed in humans to address the potential systemic effects of this oil.
